# Data on spectra of lanthanide-containing polyoxometalate

**DOI:** 10.1016/j.dib.2020.105245

**Published:** 2020-02-05

**Authors:** Chunxiang Li, Qinyu Yan

**Affiliations:** School of Chemistry and Chemical Engineering, Harbin Institute of Technology, Harbin, 150001, China

**Keywords:** Polyoxometalate, Lanthanide-containing polyoxometalate, UV–vis spectrum, IR spectrum, Cyclic voltammetric

## Abstract

Lanthanide-containing polyoxometalate (POM) is one of the presently most active groups as the properties of POM can be modified by incorporated transition metal ions. There are several reported transition metal ion contained POMs such as one Ln ion-POM with Keggin POM frameworks and a novel sandwich”-type, which consists of two open Wells-Dawson anions and three Ln ions [1–3]. In this data article, UV–vis spectra and FTIR spectra as well as the elemental analysis are shown for prepared β-K_7_H_8_[Er_3_O_3_(SiW_9_O_34_)_2_]·25H_2_O and β-K_12_H_5_[Sm_3_O_3_(SiW_9_O_34_)_2_]·18H_2_O, these results are intended to provide support for lanthanide-containing POM due to the lack of information on lanthanide-containing POM. The UV–vis spectra and FTIR spectra as well as the elemental analysis are shown for prepared β-K_7_H_8_[Er_3_O_3_(SiW_9_O_34_)_2_]·25H_2_O and β-K_12_H_5_[Sm_3_O_3_(SiW_9_O_34_)_2_]·18H_2_O were not reported in the article [4] are given. The data is related to the research article “Enhancing Electrofluorochromic Efficiency through ${\text{C}}_{30}{\text{H}}_{31}{{\text{N}}_{6}}^{+}$-Sensitized Layer-by-layer Polyoxometalate Films” [4].

**Table:** Specifications Table

Subject	Materials Chemistry
Specific subject area	Polyoxometalate (POM), a novel type of inorganic metal-oxygen cluster compounds, are attractive for applications in different fields such as catalysis, medicine, analysis, materials science, biochemistry.
Type of data	Figure
How data were acquired	Date were obtained by U-3010 UV–vis spectrophotometer, Perkin Elmer 16 PC FT-IR spectrometer, Perkin-Elmer 2400 elemental analyzer and CHI852D electrochemical workstation.
Data format	Raw, analyzed and descriptive data.
Parameters for data collection	A conventional three-electrode system was used for the electrochemical measurements, with a bare glassy carbon electrode (GCE) or a multilayer film coated GCE as a working electrode, a commercial Ag/AgCl as reference electrode and a twisted platinum wire as counter electrode. The scan rate was 50 mV s^−1^.
Description of data collection	UV–vis spectra for β-K_7_H_8_[Er_3_O_3_(SiW_9_O_34_)_2_]·25H_2_O, β-K_12_H_5_[Sm_3_O_3_(SiW_9_O_34_)_2_]·18H_2_O and Janus green in water; IR spectra for β-K_7_H_8_[Er_3_O_3_(SiW_9_O_34_)_2_]·25H_2_O and β-K_12_H_5_[Sm_3_O_3_(SiW_9_O_34_)_2_]·18H_2_O; Cyclic voltammetric analyzer for β-K_7_H_8_[Er_3_O_3_(SiW_9_O_34_)_2_]·25H_2_O and β-K_12_H_5_[Sm_3_O_3_(SiW_9_O_34_)_2_]·18H_2_O in 0.5 M acetate buffer (pH = 4.7) solutions.
Data source location	School of Chemistry and Chemical Engineering, Harbin Institute of Technology, Harbin, 150001, China
Data accessibility	Data are available in this article.
Related research article	[4] C.X. Li, Q.Y. Yan, H. Liu, B.S. Liu, Enhancing Electrofluorochromic Efficiency through ${\text{C}}_{30}{\text{H}}_{31}{{\text{N}}_{6}}^{+}$-Sensitized Layer-by-layer Polyoxometalate Films. Applied Surface Science, 503 (2020) 144321–144327.

**Table:** 

**Value of the Data** • This data will be useful for the scientists working in the area of lanthanide-containing polyoxometalate [[1], [2], [3]]. • The spectra ananlysis of lanthanide-containing polyoxometalate will be helpful to understand the structure of complexes during layer-by-layer fabrication processes. • The spectra ananlysis and the electrochemical measurements will be useful for others to compare their results. • The spectra ananlysis of lanthanide-containing polyoxometalate will be helpful for material chemists to understand the spectral interactions when the hybrid films prepared by layer-by-layer method.

## 1 Data

**Fig. 1:**
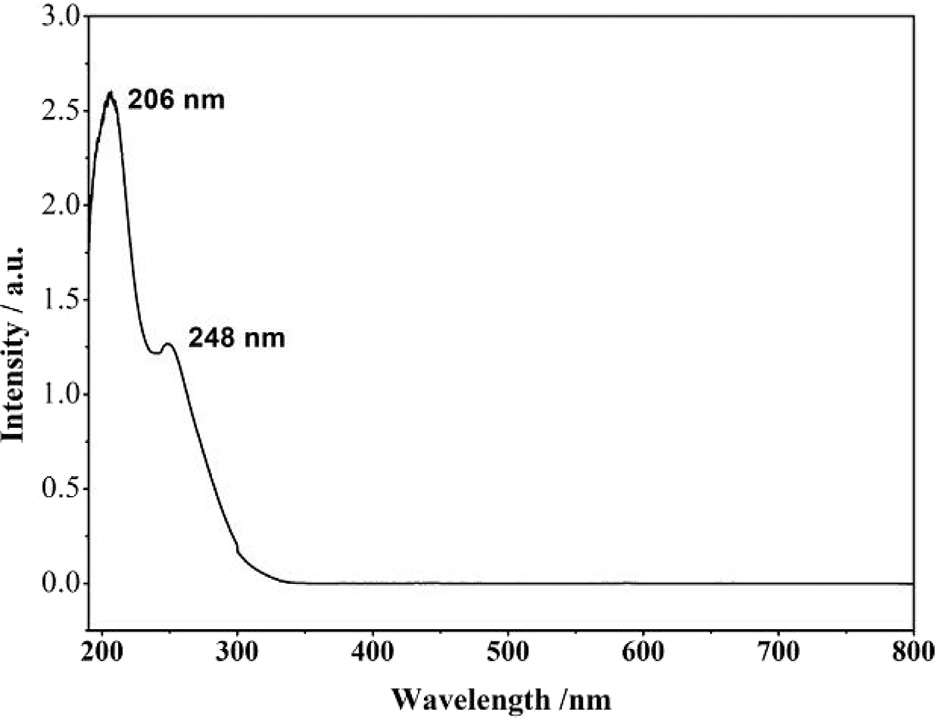
UV–vis spectrum of 2mM β-K_12_H_5_[Sm_3_O_3_(SiW_9_O_34_)_2_]·18H_2_O solution.

**Fig. 2:**
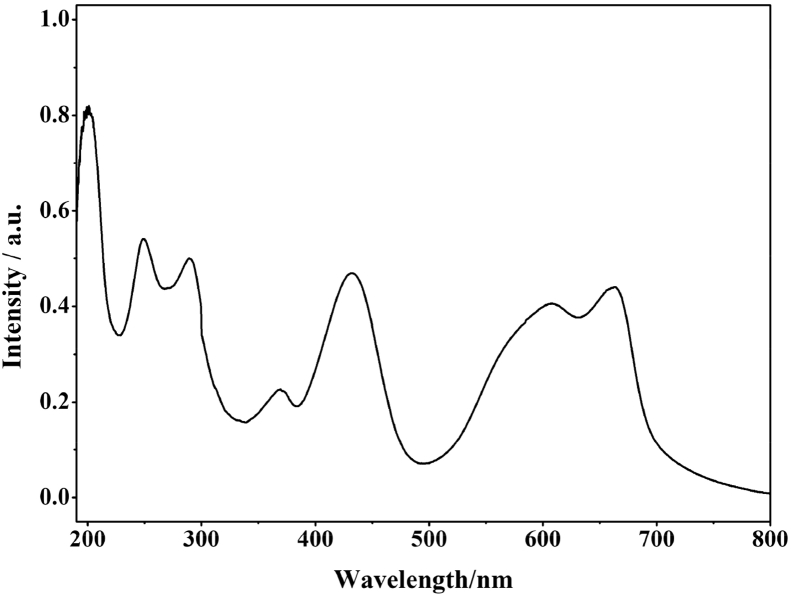
UV–vis spectrum of C_30_H_31_N_6_Cl in water.

**Fig. 3:**
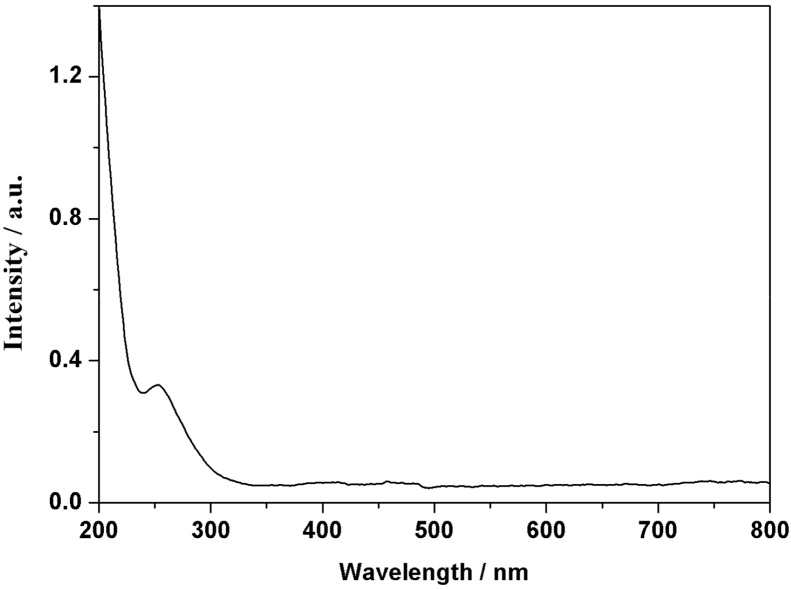
UV–vis spectrum of 2mM β-K_7_H_8_[Er_3_O_3_(SiW_9_O_34_)_2_]·25H_2_O solution.

**Fig. 4:**
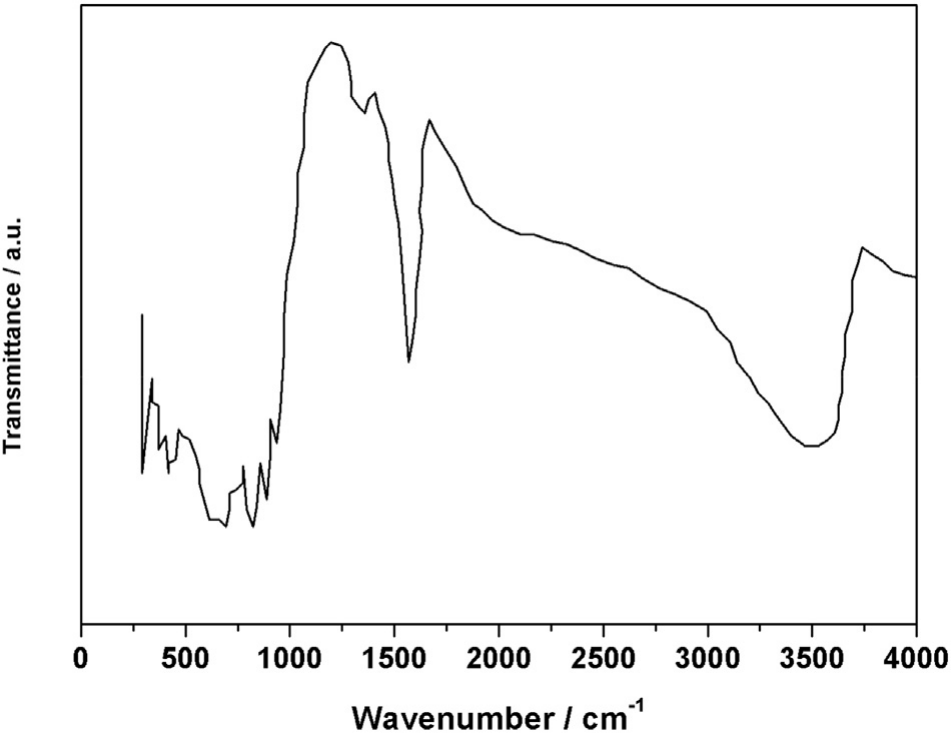
FTIR features of β-K_12_H_5_[Sm_3_O_3_(SiW_9_O_34_)_2_]·18H_2_O.

**Fig. 5:**
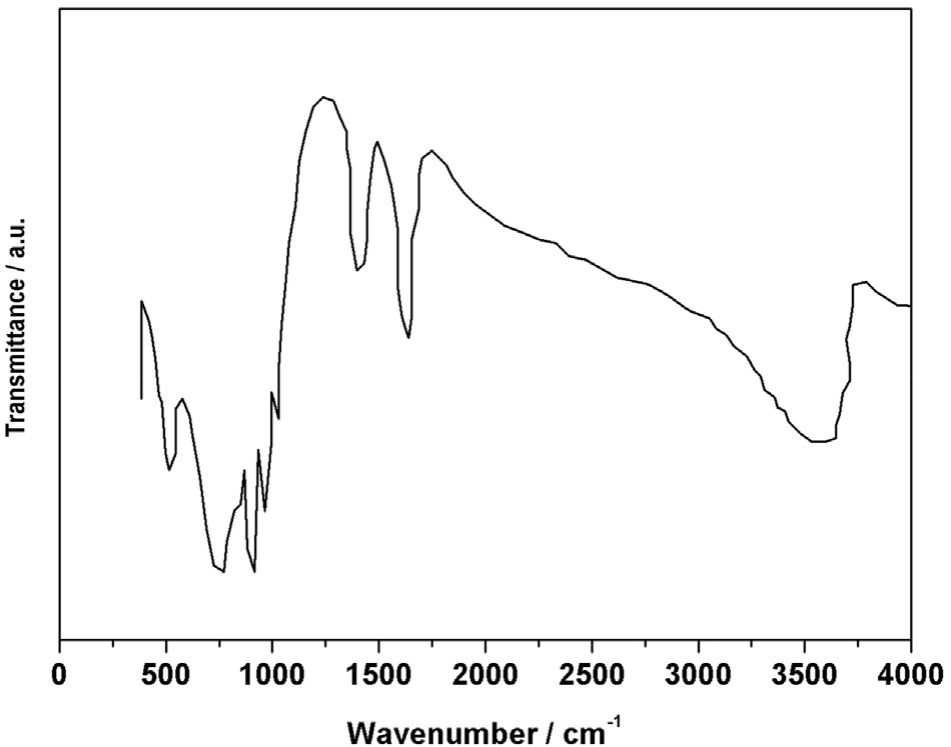
FTIR features of β-K_7_H_8_[Er_3_O_3_(SiW_9_O_34_)_2_]·25H_2_O.

**Fig. 6:**
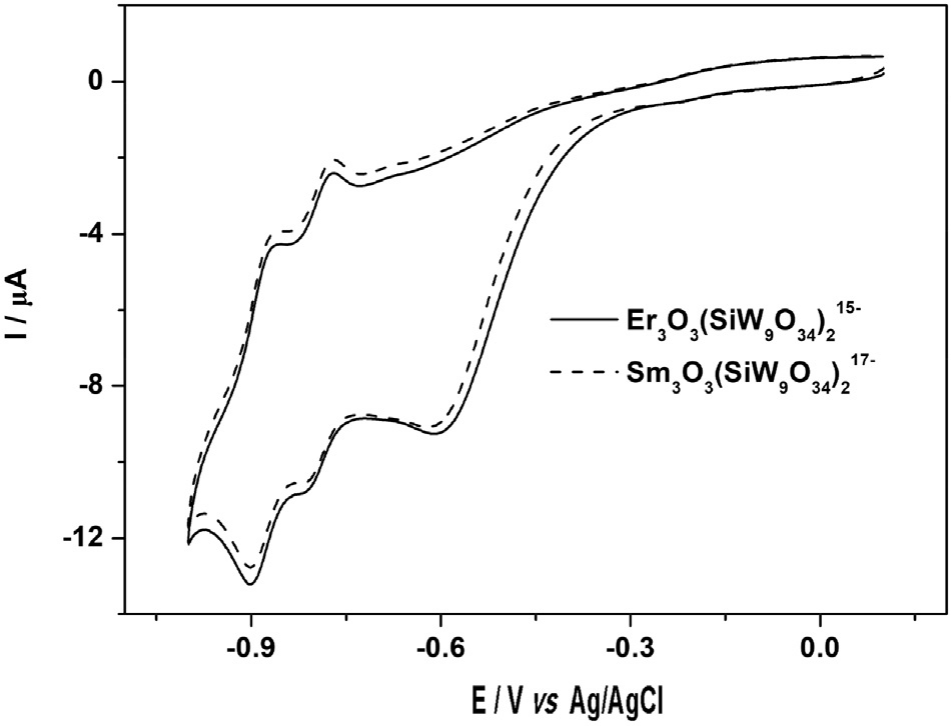
Cyclic voltammetries of 2 mM Er_3_O_3_${{\left({\text{SiW}}_{9}{\text{O}}_{34}\right)}_{2}}^{15-}$ and Sm_3_O_3_${{\left({\text{SiW}}_{9}{\text{O}}_{34}\right)}_{2}}^{17-}$ in 0.5 M acetate buffer (pH = 4.7) solutions. The scan rate was 50 mV s^−1^.

Fig. 1 shows the UV–vis spectrum of 2mM β-K_12_H_5_[Sm_3_O_3_(SiW_9_O_34_)_2_]·18H_2_O solution. Fig. 2 depicts seven absorbances for C_30_H_31_N_6_Cl in water. Fig. 3 contains data of 2mM β-K_7_H_8_[Er_3_O_3_(SiW_9_O_34_)_2_]·25H_2_O solution. Fig. 4 presents FTIR features of β-K_12_H_5_[Sm_3_O_3_(SiW_9_O_34_)_2_]·18H_2_O. Fig. 5 displays FTIR features of β-K_7_H_8_[Er_3_O_3_(SiW_9_O_34_)_2_]·25H_2_O. Fig. 6 includes two experiments made for 2mM β-K_7_H_8_[Er_3_O_3_(SiW_9_O_34_)_2_]·25H_2_O and β-K_12_H_5_[Sm_3_O_3_(SiW_9_O_34_)_2_]·18H_2_O of electrochemical measurements at the same operational conditions. The experimental conditions were: room temperature, 0.5 M acetate buffer (pH = 4.7), the scan rate is 50 mV s^−1^.

## 2 Experimental design, materials, and methods

The Er and Sm-containing silicotungstate β-K_7_H_8_[Er_3_O_3_(SiW_9_O_34_)_2_]·25H_2_O (abbr. ErSiW_9_) and β-K_12_H_5_[Sm_3_O_3_(SiW_9_O_34_)_2_]·18H_2_O (abbr. SmSiW_9_) were prepared in 0.5 M acetate buffer (pH = 5.0) according to the literature methods [5,6], the pH of the buffer solution was adjusted to 5.7 by 0.5 M NaoH during the fabrication processes. All products were recrystallized in warm water (40 °C). 2mM β-K_7_H_8_[Er_3_O_3_(SiW_9_O_34_)_2_]·25H_2_O and β-K_12_H_5_[Sm_3_O_3_(SiW_9_O_34_)_2_]·18H_2_O solutions were prepared by dissolving 2 × 10^−5^ mol cystal in 10 mL of deionized water with 18 MΩcm of resistivity.

The synthesized β-K_7_H_8_[Er_3_O_3_(SiW_9_O_34_)_2_]·25H_2_O and β-K_12_H_5_[Sm_3_O_3_(SiW_9_O_34_)_2_]·18H_2_O were characterized by U-3010 UV–vis spectrophotometer and Fourier Transform Infrared (FTIR), the FTIR spectra were recorded with PerkinElmer 16 PC FT-IR spectrometer, in a range between 300 and 4000 cm^−1^. Cyclic voltammetry was carried out in a three compartment cell (10 mL) with a CHI852D voltammetric analyzer at ambient temperature (20 ± 2 °C). All potentials are given with respect to a commercial Ag/AgCl reference electrode. A twisted platinum wire was used as the counter electrode and a glassy carbon electrode (GCE) as the working electrode. The GCEs were pre-treated by polishing with 1.0 and 0.3 μm α-Al_2_O_3_ powders, and sonicating in water for about 5 min after each polishing step. Elemental analysis was conducted on a PerkinElmer 2400 elemental analyzer.
